# Nutritional support and immunonutrition in esophageal cancer—From perioperative care to long-term survivorship: A review

**DOI:** 10.17305/bb.2025.13328

**Published:** 2025-12-02

**Authors:** Mohamed Hussein, Tayfor Mohamed, Fatma Abdulla Mubarak, Fatmah Morad, Maryam Naser, Ayesha Humaira, Haala Amina, Manal Khan

**Affiliations:** 1 Department of Biomedical Sciences, Dubai Medical College for Girls, Dubai Medical University, Dubai, UAE

**Keywords:** Esophageal cancer, nutritional therapy, immuno-nutrition, enteral nutrition, parenteral nutrition, prehabilitation, precision nutrition, quality of life, oncological care

## Abstract

Esophageal cancer is recognized as one of the most aggressive malignancies within the digestive system, with global survival rates typically falling below 20%. Malnutrition impacts up to 80% of patients, significantly affecting treatment tolerance, postoperative outcomes, and overall quality of life. Recent advancements in clinical nutrition and immunometabolism have transformed the perception of nutrition from a mere supportive measure to a vital therapeutic component in cancer care. This review synthesizes evidence from studies published between 2010 and 2025, exploring the effects of various nutritional strategies—including enteral, elemental, parenteral, immuno-nutritional, behavioral, and prehabilitative interventions—on metabolism, immune response, and clinical outcomes in patients with esophageal cancer. The findings demonstrate that targeted approaches such as immune-enhancing enteral formulations, omega-3-enriched parenteral nutrition, and structured dietary counseling can mitigate inflammation, preserve muscle mass, enhance treatment adherence, and improve psychological well-being. Overall, the literature supports the perspective of nutrition as a precision-based, integral component of multidisciplinary cancer management. Incorporating nutritional optimization throughout all stages of care—ranging from prehabilitation and perioperative support to survivorship and palliative management—can enhance metabolic resilience, promote faster recovery, and significantly improve the quality of life for individuals diagnosed with esophageal cancer.

## Introduction

Esophageal cancer continues to pose a significant global health challenge and is recognized as one of the most aggressive malignancies of the gastrointestinal tract. According to Global Cancer Observatory (GLOBOCAN 2020), it ranks as the seventh most common cancer and the sixth leading cause of cancer-related mortality worldwide, with approximately 630,000 new cases and over 500,000 deaths annually [1]. The two primary histological types, esophageal squamous cell carcinoma (ESCC) and esophageal adenocarcinoma (EAC), exhibit distinct epidemiological and etiological characteristics; however, both are associated with poor prognoses and high recurrence rates [2]. Despite advancements in surgical techniques, perioperative management, and systemic therapies, the five-year survival rate rarely exceeds 20%, highlighting the urgent need for multidisciplinary strategies aimed at improving clinical outcomes and patient well-being.

Current oncological management of esophageal cancer employs multimodal strategies that integrate systemic therapy with surgery and radiotherapy. Standard regimens typically include neoadjuvant chemoradiotherapy, often utilizing platinum–fluoropyrimidine combinations, along with perioperative chemotherapy protocols such as fluorouracil, leucovorin, oxaliplatin, and docetaxel (FLOT) for adenocarcinoma. Immunotherapy has emerged as a crucial component of contemporary care, with programmed cell death protein 1 (PD-1) inhibitors like nivolumab and pembrolizumab now incorporated into both adjuvant and advanced-stage treatment regimens. Targeted therapies, such as trastuzumab for human epidermal growth factor receptor 2 (HER2)-positive tumors, are also employed for selected patients. While these advancements have improved oncological control, they are accompanied by significant toxicities, metabolic stress, and treatment-related functional decline, underscoring the necessity for integrated supportive strategies. Within this multimodal framework, nutrition plays a pivotal role in enhancing treatment tolerance, preserving physiological reserve, and promoting overall recovery [3].

Among the various factors influencing outcomes, malnutrition is one of the most prevalent and modifiable. It affects up to 80% of patients at diagnosis and results from multiple contributing factors, including dysphagia, odynophagia, anorexia, and tumor-related metabolic dysfunction [4]. This catabolic state leads to progressive weight loss, muscle wasting, and systemic inflammation, culminating in cancer cachexia—a complex metabolic syndrome characterized by involuntary loss of skeletal muscle and functional decline [5]. Malnutrition and cachexia exacerbate treatment-related toxicities, impair immune competence, delay wound healing, and diminish quality of life. Evidence consistently demonstrates that poor nutritional status is an independent predictor of increased morbidity, higher rates of postoperative complications, prolonged hospitalization, and reduced survival [6].

Historically, nutritional management in esophageal cancer was regarded as supportive care, primarily aimed at preventing malnutrition or maintaining weight [7]. However, advancements in clinical nutrition, immunometabolism, and perioperative oncology have redefined nutrition as an active therapeutic component capable of influencing metabolic regulation, inflammatory control, and immune function [8]. Contemporary research indicates that nutritional intervention can modulate treatment tolerance, enhance metabolic stability, and accelerate recovery, positioning it as a critical pillar within precision oncology [9].

Targeted nutritional interventions encompass a wide range of strategies, from elemental and disease-specific enteral formulas designed to enhance nutrient absorption and modulate inflammation, to immuno-nutrition that incorporates nutrients such as omega-3 fatty acids, arginine, glutamine, and nucleotides to support immune recovery and tissue repair [10]. Parenteral nutrition, particularly formulations enriched with fish oil, plays a vital role during periods of gastrointestinal compromise or postoperative catabolic stress [11]. Furthermore, the concept of prehabilitation, which combines nutritional optimization with physical and respiratory conditioning prior to therapy, has emerged as a proactive strategy to build physiological resilience, enhance treatment tolerance, and improve postoperative outcomes [12]. In advanced disease stages, the focus of nutritional care shifts toward comfort, autonomy, and dignity, emphasizing individualized, compassionate approaches that align with patients’ goals and values [13].

**Figure 1.**
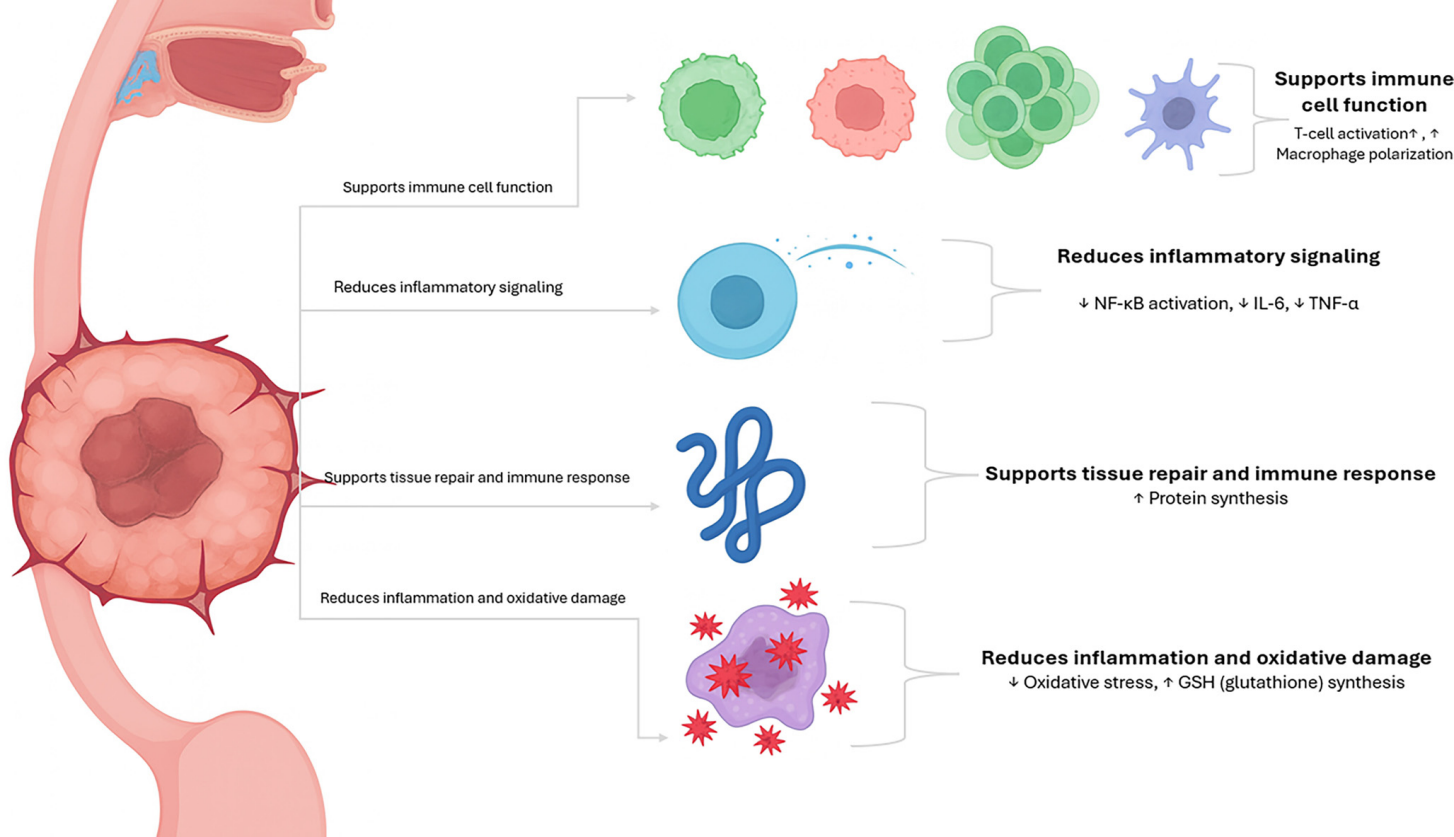
**Schematic overview of nutrient-mediated modulation of immune and metabolic pathways in esophageal cancer.** Essential nutrients, including omega-3 fatty acids, arginine, and glutamine, facilitate immune cell activation, attenuate inflammatory cytokine signaling, enhance antioxidant defenses, and promote tissue repair and protein synthesis.

**Figure 2.**
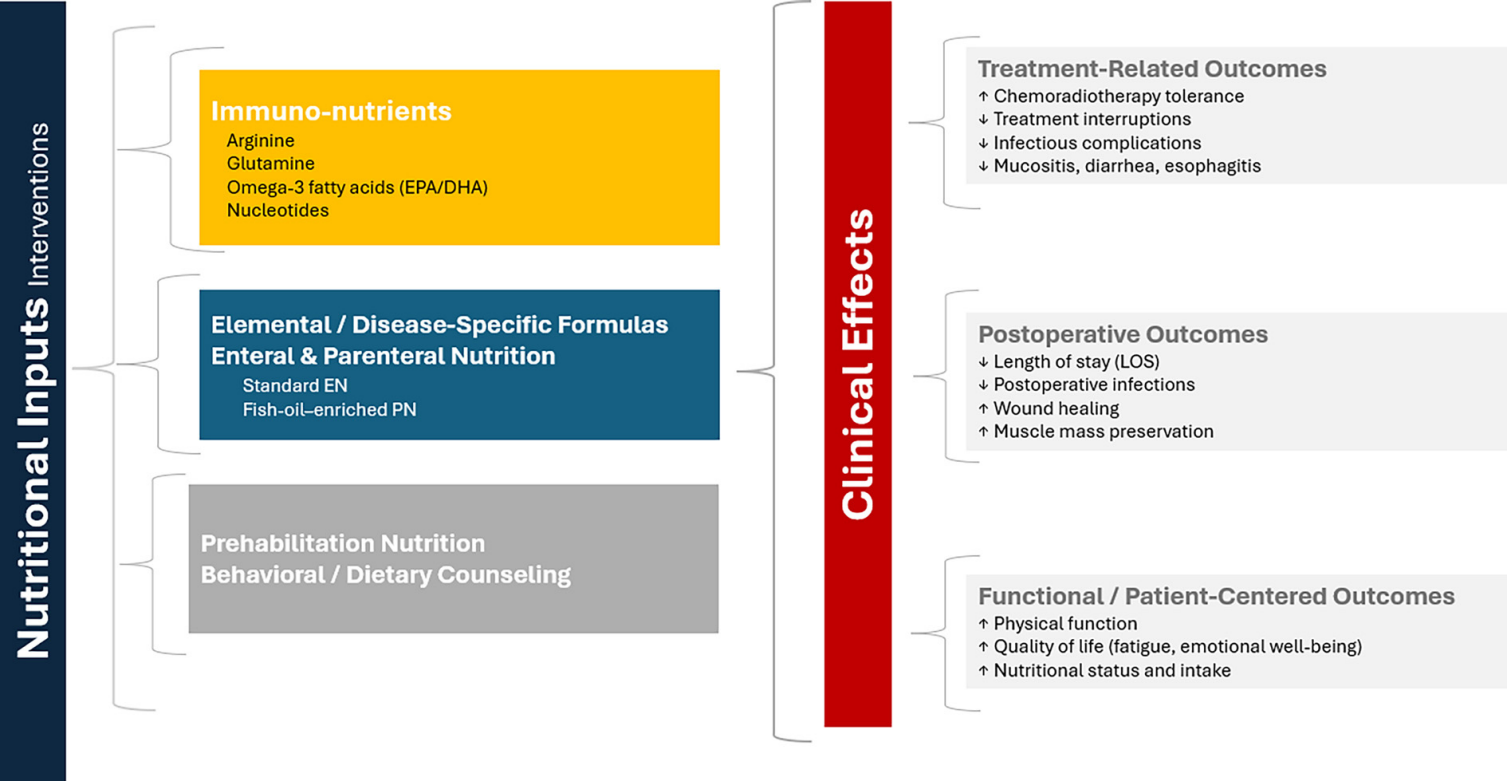
**Overview of the impact of nutritional interventions on outcomes in esophageal cancer.** Nutritional strategies, such as immunonutrients, specialized formulas, enteral and parenteral nutrition, prehabilitation, and dietary counseling, enhance clinical outcomes. These interventions lead to improved treatment tolerance, reduced complications, shorter postoperative recovery times, and superior functional and patient-reported outcomes. Abbreviations: EPA: Eicosapentaenoic acid; DHA: Docosahexaenoic acid.

Despite the growing body of evidence, research in this field remains fragmented. Studies vary widely in sample size, intervention design, nutrient composition, and outcome measures, complicating the establishment of standardized clinical guidelines [14]. Many trials emphasize surrogate markers, such as body weight or serum albumin, rather than comprehensive patient-reported or biomarker-based outcomes, and few investigate long-term survivorship benefits [15]. These limitations underscore the need for integrative reviews that bridge clinical, metabolic, and psychosocial dimensions of nutrition in esophageal cancer (Figures 1 and 2). To address these gaps, this review provides a comprehensive synthesis of nutritional strategies in esophageal cancer, evaluating their effects on treatment outcomes, metabolic balance, immune recovery, and overall quality of life. The review further explores the biological mechanisms by which targeted nutrients influence inflammation, metabolism, and immunity while identifying significant research gaps and methodological inconsistencies, including variability in study design, lack of standardized endpoints, and limited integration of metabolic and molecular biomarkers. Finally, it introduces a precision-nutrition framework that integrates metabolic profiling, inflammatory markers, and patient-centered outcomes to guide personalized nutritional therapy.

## Materials and methods

A comprehensive literature search was conducted using the PubMed database, selected for its extensive indexing of peer-reviewed biomedical and clinical research pertinent to oncology and nutrition. The search strategy utilized the Boolean string “Nutritional Interventions” AND “Esophageal Cancer,” applying additional filters and Medical Subject Headings (MeSH) terms to identify studies focused on nutritional management in esophageal cancer. The search encompassed the period from January 2010 to October 2025, ensuring the inclusion of contemporary advances in perioperative nutrition, immuno-nutrition, and supportive oncologic care.

The objective of this review was to provide a clinically oriented synthesis rather than a quantitative meta-analysis, integrating trial outcomes and mechanistic insights from diverse sources. Consequently, while the search initially targeted interventional studies, including randomized controlled trials, clinical trials, and prospective cohort studies, it was intentionally broadened to encompass systematic reviews, meta-analyses, and evidence-based clinical guidelines (e.g., the European Society for Clinical Nutrition and Metabolism [ESPEN] and the National Comprehensive Cancer Network [NCCN]) when these sources offered data or consensus statements directly relevant to esophageal cancer. Additionally, mixed-population studies involving patients with upper gastrointestinal or head-and-neck cancers were included if they reported subgroup data for esophageal cancer or if their findings were scientifically generalizable to similar treatment and nutritional contexts. This broader inclusion aimed to ensure a comprehensive and clinically meaningful perspective on nutritional therapy within multidisciplinary cancer care.

Studies were deemed eligible if they investigated any form of nutritional intervention, including enteral or parenteral nutrition, disease-specific or immune-enhancing formulas, amino acid or fatty acid supplementation, structured dietary counseling, or multimodal prehabilitation incorporating nutrition. Publications focusing solely on animal or *in vitro* work, narrative commentaries without original data, or case reports and conference abstracts were excluded. Studies were also excluded if they lacked measurable clinical outcomes, such as nutritional status, inflammatory or immune biomarkers, postoperative complications, quality of life, or survival. All retrieved articles were screened by title and abstract to assess relevance, followed by full-text evaluation for potentially eligible studies. Key information, including study design, patient population, intervention type, comparator, timing, outcome measures, and principal findings, was extracted and summarized. This methodological approach provides a transparent and inclusive framework that integrates both direct clinical evidence from interventional studies and contextual evidence from guidelines and higher-level syntheses. It reflects how nutritional therapy is currently evaluated and applied in modern esophageal cancer management, recognizing that clinical practice often draws from overlapping evidence across related gastrointestinal tumor types.

## Results and discussion

The role of nutrition in esophageal cancer management has increasingly evolved from a supportive adjunct to a fundamental component of therapeutic strategy. Malnutrition remains one of the most prevalent yet modifiable determinants of treatment tolerance, postoperative recovery, and overall quality of life in this patient population. Evidence synthesized in this review indicates that nutritional interventions—including patient education, enteral and parenteral nutrition, immuno-nutrition, and prehabilitation—function as active therapeutic modalities capable of influencing metabolic regulation, immune function, and clinical outcomes. Drawing upon clinical and interventional studies published between 2010 and 2025, this discussion delineates the multifaceted contributions of nutrition across the continuum of esophageal cancer care, encompassing preoperative optimization, adjuvant treatment, palliative management, and survivorship. Collectively, current findings highlight a paradigm shift in oncologic nutrition, redefining nutritional support from reactive correction of deficiency to proactive, precision-based therapy that integrates metabolic, immunologic, and psychosocial dimensions of recovery (see Table 1 and Table S1).

**Table 1:** Randomized controlled trials of nutritional interventions in esophageal cancer

**Study (author, year)**	**Sample size (*n*)**	**Intervention/comparator**	**Timing**	**Primary endpoint**	**Key result (summary)**	**Risk of bias/notes**
Pinto et al., 2021 (QOLEC2) [8]	143	Nutritional + respiratory counseling vs standard care	Post-esophagectomy	Functional recovery (QoL score)	Improved physical function (+6 points, *P* < 0.05); no difference in readmissions	Single-center, limited blinding
Poulsen et al., 2014 [17]	150	Individualized dietary counseling vs general advice	During treatment	Body weight and energy intake	Improved adherence and less fatigue; no effect on survival	Mixed cancer types; esophageal subgroup small
Vasson et al., 2014 [13]	115	Immunonutrition vs standard enteral diet	During chemoradiotherapy	Functional capacity	Maintained albumin and performance scores (*P* < 0.05)	Mixed head and neck + esophageal population
Kitagawa et al., 2017 [19]	64	Immunomodulating diet vs standard EN	Preoperative	Infection rate	Lower infections (15% vs 38%, *P* ═ 0.03); improved immune markers	Small, single-center RCT
Chitapanarux et al., 2020 [20]	80	Arginine + glutamine + fish oil vs standard support	Concurrent chemoradiotherapy	Inflammation and mucosal toxicity	Reduced mucositis and better treatment tolerance	Modest sample; short follow-up
Long et al., 2013 [21]	60	Fish-oil-enriched PN vs standard PN	Postoperative	Inflammatory cytokines (IL-6, TNF-α)	Lower cytokine levels and improved immune cell function	Single-center, unblinded
Katada et al., 2021 (KDOG 1101) [22]	120	Amino-acid-rich elemental diet vs standard care	During chemotherapy	Grade ≥ 2 GI toxicity	Lower diarrhea rate (18% vs 35%, *P* ═ 0.04)	Unblinded; short follow-up
Tanaka et al., 2016 [23]	30	Elemental diet + glutamine vs control	Chemotherapy	Mucositis severity	Trend to reduced mucositis (*P* ═ 0.07)	Feasibility study; underpowered
Ishikawa et al., 2016 [25]	40	Elemental diet vs standard diet	Chemoradiotherapy	Lean body mass	Preserved muscle (+0.2 kg/m^2^ vs −0.8 kg/m^2^, *P* < 0.05)	Small sample; short duration
St-Pierre et al., 2024 [14]	52	Multimodal prehabilitation vs usual care	Neoadjuvant phase	VO_2_ max change	↑ Cardiorespiratory fitness (+1.8 mL/kg/min, *P* ═ 0.02)	Feasibility trial
Chen et al., 2025 [27]	86	Walking + diet education vs standard care	Rehabilitation	QoL (EORTC QLQ-C30)	Improved fatigue and social function (*P* < 0.05)	Single-center; no compliance tracking
Fietkau et al., 2013 [28]	173	Disease-specific EN vs standard EN	Chemoradiotherapy	Body weight	Maintained weight (−0.4 kg vs −2.1 kg, *P* < 0.01)	Mixed head and neck + esophageal cases
Aiko et al., 2012 [29]	60	Antioxidant-enriched IED vs control	Perioperative	Post-op infection rate	Reduced infections (13% vs 33%, *P* ═ 0.04)	Open-label; single institution
Markar et al., 2025 (NEOIMMUNE) [30]	258 (total); 92 esophageal	Immunonutrition vs standard EN	Neoadjuvant/Perioperative	QoL change at 12 wk	Small improvement in fatigue and function (*P* ═ 0.03)	Multicenter; short follow-up

Abbreviations: QoL: Quality of life; EN: Enteral nutrition; PN: Parenteral nutrition; GI: Gastrointestinal; IL-6: Interleukin-6; TNF-α: Tumor necrosis factor-alpha; VO_2_ max: Maximal oxygen uptake; EORTC QLQ-C30: European Organisation for Research and Treatment of Cancer Quality of Life Questionnaire-Core 30; IED: Immuno-enhanced diet; RCT: Randomized controlled trial.

### The burden of malnutrition and rationale for nutritional optimization

Malnutrition is a critical and modifiable determinant of outcomes in esophageal cancer, affecting approximately 60%–80% of patients. It contributes to postoperative complications, immune suppression, prolonged hospitalization, and reduced survival rates. Dysphagia, anorexia, and treatment-induced catabolism lead to significant nutritional depletion well before therapy begins. In the QOLEC2 randomized controlled trial, Pinto et al. [16] evaluated 143 patients who underwent esophagectomy, assigning them to either a structured program of nutritional and respiratory counseling or standard follow-up care. The primary endpoint was functional recovery, assessed using validated quality-of-life scales. The intervention group demonstrated modest yet significant improvements in physical performance (mean difference +6 points, *P* < 0.05) and fatigue scores, although readmission rates remained unchanged. The authors noted limited blinding and a single-center design as key limitations.

Similarly, Poulsen et al. [17] assessed individualized nutritional counseling in 150 cancer patients, including those with esophageal malignancy. The primary endpoint focused on weight maintenance and energy intake. Personalized counseling improved dietary adherence and reduced treatment-related fatigue, though overall survival remained unaffected. Collectively, these studies underscore the clinical importance of early, structured nutritional counseling. While improvements in function and quality of life are consistently observed, long-term survival benefits remain uncertain due to small sample sizes and heterogeneous study designs. More recently, Terayama et al. [18] investigated the ENLICHE study, exploring a low-carbohydrate enteral nutrition formula to mitigate postoperative hyperglycemia in non-diabetic patients following esophagectomy. Their results indicated that stabilizing blood glucose levels post-surgery was associated with fewer infections and shorter hospital stays, highlighting the growing significance of precise metabolic nutrition over mere calorie replacement.

### Immuno-nutrition: Mechanistic insights and clinical benefits

Immuno-nutrition transcends basic nutrient replacement by actively influencing the body’s immune and inflammatory responses. Formulas enriched with arginine, glutamine, and omega-3 fatty acids have demonstrated meaningful biological and clinical improvements. Arginine enhances T-cell functionality and nitric oxide production, thereby strengthening immune defense. Glutamine acts as a crucial energy source for rapidly dividing cells and facilitates antioxidant protection through the synthesis of glutathione. Additionally, omega-3 fatty acids, including eicosapentaenoic acid (EPA) and docosahexaenoic acid (DHA), contribute to the reduction of inflammation by downregulating cytokine activity driven by nuclear factor kappa-light-chain-enhancer of activated B cells (NF-κB). In a randomized clinical trial involving 115 patients with head-and-neck or esophageal cancers, Vasson et al. [13] compared an immune-enhancing enteral formula to an isocaloric standard diet during chemoradiotherapy. The primary endpoint was functional capacity, with secondary endpoints including infection rate and treatment tolerance. Patients receiving the enriched formula maintained higher serum albumin levels and better performance scores (*P* < 0.05), although no differences in survival were observed. Limitations included the mixed tumor population and the absence of stratified randomization. In another prospective randomized trial, Kitagawa et al. [19] enrolled 64 esophageal cancer patients to evaluate preoperative immunonutrition vs a standard formula, focusing on the postoperative infection rate. The immune-enhanced diet reduced infectious complications (15% vs 38%, *P* ═ 0.03) and improved immune markers, though mortality outcomes were not significantly affected. Similarly, Chitapanarux et al. [20] conducted a randomized controlled study assessing supplementation with arginine, glutamine, and fish oil during concurrent chemoradiotherapy. The intervention group exhibited lower inflammatory and mucosal toxicity scores and improved treatment adherence compared to standard nutritional support. Long et al. [21] investigated postoperative parenteral nutrition enriched with fish oil in patients undergoing esophagectomy, reporting reduced circulating levels of pro-inflammatory cytokines, including IL-6 and tumor necrosis factor alpha (TNF-α), alongside modest improvements in immune-cell activity. Collectively, these findings suggest that targeted nutrient combinations may beneficially modulate inflammatory and immune responses, offering physiological advantages beyond those achieved through conventional nutrition alone, although larger, multi-center studies are needed to validate these effects.

### Elemental diets and gastrointestinal toxicity

Patients with esophageal cancer undergoing intensive chemotherapy often experience severe gastrointestinal side effects, such as mucositis, diarrhea, and loss of appetite, which can impede nutrient absorption and hinder the maintenance of proper nutrition. To address these challenges, several Japanese studies have explored the use of elemental diets, consisting of pre-digested amino acids that are more readily absorbed by the body, even when digestive function is compromised. The KDOG 1101 trial by Katada et al. [22] involved 120 participants, with the primary endpoint being the incidence of grade ≥ 2 gastrointestinal toxicities. The elemental diet group demonstrated a lower frequency of diarrhea (18% vs 35%, *P* ═ 0.04) and achieved better caloric maintenance. However, key limitations included the lack of blinded evaluation and a short follow-up period. Building on this, Tanaka et al. [23] assessed the combination of an elemental diet and glutamine supplementation in 30 patients with esophageal cancer, with the primary endpoint being the severity of oral mucositis. The intervention indicated a non-significant trend toward reduced mucosal injury (*P* ═ 0.07) without affecting chemotherapy response. A subsequent erratum confirmed the consistency of the reported data. Most recently, in the EPOC2 phase III trial [24], it was found that elemental diets further mitigated the severity of chemotherapy-related side effects without compromising treatment effectiveness. Complementing these findings, Ishikawa et al. [25] examined the same amino acid-based elemental formula in 40 patients undergoing chemo- or chemoradiotherapy, with the primary endpoint being the preservation of lean body mass. Patients in the intervention arm maintained a skeletal muscle index (+0.2 kg/m^2^ vs. −0.8 kg/m^2^, *P* < 0.05). The authors identified the small sample size and limited duration of follow-up as important limitations.

### Prehabilitation and functional optimization

Patients with esophageal cancer frequently encounter considerable fatigue and physical decline due to prolonged treatment regimens and persistent inflammation. Recent studies underscore the efficacy of multimodal prehabilitation—programs that integrate nutrition, exercise, and psychological support—to enhance patients’ strength prior to surgery and bolster their overall resilience. St-Pierre et al. [26] assessed the effects of a multimodal prehabilitation program on 52 patients with esophageal cancer undergoing neoadjuvant chemotherapy. Participants were randomized to either the structured program or standard preoperative care. The primary outcome measure was the change in peak oxygen uptake (VO_2_ max), while secondary outcomes included muscle strength and quality-of-life indices. The intervention resulted in significant enhancements in cardiorespiratory fitness (+1.8 mL/kg/min, *P* ═ 0.02) and handgrip strength, although no significant differences in short-term quality-of-life scores were observed between groups. The authors noted the limited sample size and short follow-up duration as significant constraints. Similarly, Chen et al. [27] involved 86 patients who participated in a three-month structured walking and dietary education program compared to usual care. The primary endpoint was global quality of life, assessed using the EORTC QLQ-C30 questionnaire at 12 months. Participants in the intervention group reported sustained improvements in fatigue, social functioning, and emotional well-being (*P* < 0.05). However, the lack of objective adherence monitoring and the single-center design limited the generalizability of these findings. Overall, prehabilitation programs incorporating tailored nutritional support seem to enhance physical conditioning and patient engagement prior to treatment. Nevertheless, data on long-term survival and surgical recovery remain limited, highlighting the need for larger, adequately powered multicenter trials.

### Specialized nutrition during chemoradiotherapy

In patients undergoing concurrent chemoradiotherapy, Fietkau et al. [28] investigated a disease-specific enteral nutrition formula in 173 patients with head-and-neck or esophageal cancers. The primary endpoint was weight maintenance during therapy. Patients receiving the specialized formula maintained a more stable body weight (−0.4 kg vs −2.1 kg, *P* < 0.01) and demonstrated higher functional performance scores, although no significant impact on overall survival was observed. Additionally, Aiko et al. [29] evaluated an antioxidant- and immune-enhanced perioperative formula in 60 patients with esophageal cancer following neoadjuvant treatment. The primary endpoint was the postoperative infection rate. The intervention resulted in a reduction in infectious complications (13% vs 33%, *P* ═ 0.04) but did not decrease the length of hospital stay. The authors acknowledged that the open-label, single-institution design and limited sample size restricted the broader applicability of their findings.

Collectively, these studies support the short-term benefits and clinical feasibility of disease-specific or immune-enhanced formulas during chemoradiotherapy. However, the evidence base remains heterogeneous, underscoring the necessity for standardized endpoints and patient stratification in future research.

### Large-scale and long-term evidence

Recent large-scale randomized controlled trials have significantly advanced this field. The NEOIMMUNE trial conducted by Markar et al. [30] was a double-blind, multicenter randomized study involving 258 patients, including 92 with esophageal cancer, comparing perioperative immunonutrition to a standard enteral formula. The primary endpoint was the change in global quality-of-life score at 12 weeks, with secondary endpoints including postoperative complications and nutritional biomarkers. The immunonutrition group exhibited modest but statistically significant improvements in fatigue and physical function domains (*P* ═ 0.03), although no significant differences were noted in infection rates or mortality. The investigators highlighted high inter-center variability and relatively short follow-up as major limitations. Supporting this evidence, both the EPOC2 trial by Tanaka et al. [24] and the ENLICHE study by Terayama et al. [18] confirmed that specialized enteral nutrition formulas are safe and effective in mitigating metabolic stress during treatment. In terms of long-term effects, Wang et al. [31] followed 29,584 participants over 25 years after a 5.25-year vitamin-mineral supplementation program. Although not exclusively focused on esophageal cancer, the study demonstrated a sustained reduction in overall and cancer-specific mortality (relative risk 0.89; 95% CI 0.81–0.97), suggesting potential long-term survival benefits of comprehensive nutritional optimization.

### Limitations and challenges

Despite the growing recognition of the therapeutic significance of nutrition in the management of esophageal cancer, several limitations persist that affect the quality and consistency of the current evidence. A primary challenge is the substantial variability in study designs [32]. Many clinical trials involve relatively small cohorts, utilize differing nutritional protocols, and apply various outcome measures, complicating the comparison of results and the execution of meaningful meta-analyses [33]. Additionally, disparities in tumor stage, treatment type, and patients’ baseline nutritional status further complicate data interpretation, obstructing the development of unified clinical guidelines [34].

Another limitation is the lack of standardized nutritional assessment methods. Many studies rely on basic anthropometric or biochemical indicators rather than employing validated screening tools or patient-reported quality-of-life measures such as the EORTC QLQ-C30 or QLQ-OG25 [35]. Consequently, the broader effects of nutritional interventions on long-term recovery and patient well-being remain inadequately defined. The optimal timing and individualization of nutritional therapy are also unclear. It has not yet been established when to initiate enteral, parenteral, or immuno-nutritional support—whether during neoadjuvant therapy, perioperative care, or palliative treatment [36]. Similarly, few studies adopt biomarker-based or phenotype-specific approaches that could allow for the tailoring of nutrition to patients’ metabolic and inflammatory profiles [37].

Methodological shortcomings are further exacerbated by the absence of long-term follow-up, which limits insights into survivorship outcomes or the durability of nutritional benefits beyond the treatment period [38]. Practical and economic barriers, including high costs of specialized formulas, limited access to dietetic expertise, and disparities in institutional infrastructure, further hinder widespread implementation, particularly in resource-limited settings. Finally, while emerging approaches such as prehabilitation and behavioral nutrition have demonstrated considerable promise, their integration into standard care necessitates a stronger multidisciplinary framework that includes oncologists, surgeons, dietitians, physiotherapists, and psychologists. To advance the field, future studies should prioritize large, multicenter randomized controlled trials that utilize standardized outcome measures and incorporate molecular and metabolic profiling to enhance our understanding of underlying mechanisms, refine protocols, and establish a more robust evidence base for nutritional therapy in esophageal cancer.

## Conclusion

The role of nutrition in esophageal cancer care has evolved significantly, transforming from a supportive afterthought into a central, evidence-based component of contemporary treatment. It is now evident that nutritional interventions extend beyond merely maintaining caloric intake; they can influence metabolism, enhance immune function, improve treatment tolerance, and facilitate both physical and emotional recovery. Strategies such as individualized enteral feeding, immune-enhancing formulas, omega-3-enriched parenteral nutrition, and comprehensive prehabilitation programs have demonstrated tangible benefits in patient outcomes and quality of life throughout the cancer continuum.

Thus, nutrition should be considered a dynamic, therapeutic approach that adapts to each patient’s unique biology, treatment plan, and psychological needs. Nevertheless, notable gaps in the evidence remain. Existing studies often vary in design, scale, and focus, which complicates the establishment of universal protocols and the comparison of findings across clinical settings. To strengthen the evidence base, future research must emphasize large, well-structured trials that incorporate metabolic, inflammatory, and molecular biomarkers to guide truly personalized nutritional care. Achieving this vision will necessitate close collaboration among multidisciplinary teams, including oncologists, surgeons, dietitians, psychologists, and rehabilitation specialists, all working together to integrate nutrition as a continuous and proactive element of cancer management. Ultimately, embedding personalized nutrition across all phases of care—from prevention and prehabilitation to treatment, recovery, and palliation—can enhance patients’ resilience, improve treatment efficacy, and elevate overall quality of life. In the current era of precision oncology, nutrition should no longer be viewed as merely supportive care; it must be recognized as an emerging and increasingly vital pillar of holistic, patient-centered cancer therapy.

## Supplemental data

**Table S1:** Ongoing or planned randomized trials

**Study (author, year)**	**Design/phase**	**Intervention/comparator**	**Planned *n***	**Status/notes**
Terayama et al., 2025 (ENLICHE) [8]	Phase II exploratory protocol	Low-carbohydrate EN vs standard EN	80	Protocol only—clinical outcomes pending
Cao et al., 2022 (POINT Trial) [17]	Multicenter RCT protocol	Pre-operative immunonutrition vs standard EN	200	Recruiting phase

Abbreviations: EN: Enteral nutrition; RCT: Randomized controlled trial.
